# Zn(ferulate)-LSH Systems as Multifunctional Filters

**DOI:** 10.3390/molecules26082349

**Published:** 2021-04-17

**Authors:** Gustavo Pereira Saito, Ana Carolina Lanfredi Matsumoto, Renata Pires Assis, Iguatemy Lourenço Brunetti, Marco Aurélio Cebim, Marian Rosaly Davolos

**Affiliations:** 1 Institute of Chemistry, São Paulo State University (Unesp), Araraquara 14800-060, São Paulo, Brazil; gustavopsaito@gmail.com (G.P.S.); carol.matsumoto@hotmail.com (A.C.L.M.); marco.cebim@unesp.br (M.A.C.); 2 School of Pharmaceutical Sciences, São Paulo State University (Unesp), Araraquara 14800-903, São Paulo, Brazil; renatapires_17@hotmail.com (R.P.A.); iguatemy.brunetti@unesp.br (I.L.B.)

**Keywords:** layered single metal hydroxides, hydroxycinnamic anions, intercalation, ultrasound treatment, UV protection, antioxidant activity

## Abstract

Excessive UV solar radiation exposure causes human health risks; therefore, the study of multifunctional filters is important to skin UV protective ability and also to other beneficial activities to the human organism, such as reduction of reactive oxygen species (ROS) responsible for cellular damages. Potential multifunctional filters were obtained by intercalating of ferulate anions into layered simple metal hydroxides (LSH) through anion exchange and precipitation at constant pH methods. Ultrasound treatment was used in order to investigate the structural changes in LSH-ferulate materials. Structural and spectroscopic analyses show the formation of layered materials composed by a mixture of LSH intercalated with ferulate anions, where carboxylate groups of ferulate species interact with LSH layers. UV-VIS absorption spectra and *in vitro* SPF measurements indicate that LSH-ferulate systems have UV shielding capacity, mainly UVB protection. The results of reactive species assays show the ability of layered compounds in capture DPPH^•^, ABTS^•+^, ROO^•^, and HOCl/OCl^−^ reactive species. LSH-ferulate materials exhibit antioxidant activity and singular optical properties that enable their use as multifunctional filters.

## 1. Introduction

Solar ultraviolet radiation (UV) is important for living organisms [1], especially humans [2], because it induces photochemical reactions responsible for the development and survival of these living species [3]. However, excessive UV radiation exposure causes damaging biological effects in the human organism [1,2,3,4,5,6] such as sunburns, premature aging, irregular skin pigmentation, and skin cancer. Moreover, UV radiation can be considered an occupational risk [7] due to people being exposed to sunlight throughout their lives. Therefore, sun protection is essential to minimize human health damages.

The chemical compounds that absorb and/or scatter UV radiation without changes in their physicochemical properties or do not decompose are denominated inorganic or organic filters [8]. These compounds are optical filters [9,10], which attenuate UV light and can transmit other radiations that compose solar spectrum. The UV shielding capacity of inorganic or organic filters makes them active constituents of the photoprotective products, e.g., sunscreens [11].

Organic filters are molecules that have chromophore groups [12] commonly with a high degree of π-conjugated systems. The UV absorption of organic filters involves electronic transitions between HOMO-LUMO orbitals, which often provide π→π* and/or *n*→π* transitions [3,12]. Among organic filters, hydroxycinnamic acids have been widely used in sunscreens due to UV shielding ability, low dermal toxicity, and less harmful to the environment [13,14,15]. An example of these molecules is the 3-(4-hydroxy-3-methoxyphenyl)prop-2-enoic acid (ferulic acid—C_10_H_10_O_4_). In general, hydroxycinnamic acids are organic compounds present in plant metabolism [16], which are easily obtained by extraction and separation methods of natural products [17]. A characteristic property of hydroxycinnamic acids is the antioxidant activity [18].

A problem related to organic filters is the decomposition of these compounds when subjected to certain conditions [5,19] such as temperature and oxidizing media. This decomposition changes the optical properties of the chromophore species, decreasing UV protection efficiency [20]. In this perspective, the thermo, chemical, and photochemical stability of organic filters can be increased and enhance their UV shielding capacity by intercalating them into layered simple metal hydroxides (LSH). The LSH are layered-structure materials that have metal-hydroxyl host layers with or without charge-balancing anions in the interlayer spacing [21]. One type of LSH structure is derived from brucite mineral, where a part of the hydroxide groups of brucite-like layers has been replaced by anions or water molecules [22,23]. In the substitution of hydroxyl anions by water molecules, interlayer anions must be present in the second coordination sphere in order to neutralize the positively charged layers [23]. These LSH materials, also named layered hydroxide salts [23], present themselves as having the following formula M^2+^(OH)_2−x_(A^n−^)_x/n_·zH_2_O (M^2+^ = metal ion and A^n−^ = interlayer anion). The intercalation of organic filters in LSH has been reported in the literature [24,25,26,27,28,29] due to broad-spectrum filters that were obtained having low skin irritability and thermal stability. However, the scientific studies do not show the UV shielding ability combined to antioxidant activity of LSH intercalated with organic anions.

Therefore, the study of intercalating hydroxycinnamic species in the LSH is of great significance for the development of materials with UV radiation protection, high photochemical stability, low toxicity, and antioxidant capacity, i.e., multifunctional filters. Thus, this work aims to investigate potential multifunctional filters obtained by the interaction of 3-(4-hydroxy-3-methoxyphenyl)prop-2-enoic acid (ferulic acid), in its anionic form, in the layered zinc hydroxide nitrate (Zn-NO_3_-LSH) matrix, emphasizing the spectroscopic characterization and antioxidant activity of the layered materials.

## 2. Results and Discussion

According to synthetic methods and parameters used to obtain the LSH materials, in this work, the zinc hydroxide nitrate host matrix was designated Zn-NO_3_-LSH. LSH-ferulate material synthesized by the anion exchange method was named Zn-fel-LSH/A and LSH-ferulate materials prepared by the precipitation method with Zn^2+^/C_10_H_9_O_4_^−^ = 3, 4 and 5 molar ratio were designated Zn-fel(3)-LSH, Zn-fel(4)-LSH, and Zn-fel(5)-LSH, respectively. In addition, LSH intercalated with ferulate anions synthesized by precipitation method with the Zn^2+^/C_10_H_9_O_4_^−^ = 4 molar ratio and subjected to ultrasound treatment for 10, 20, and 30 min were named Zn-fel(4)-LSH/U10, Zn-fel(4)-LSH/U20, and Zn-fel(4)-LSH/U30, respectively.

**Figure A1:**
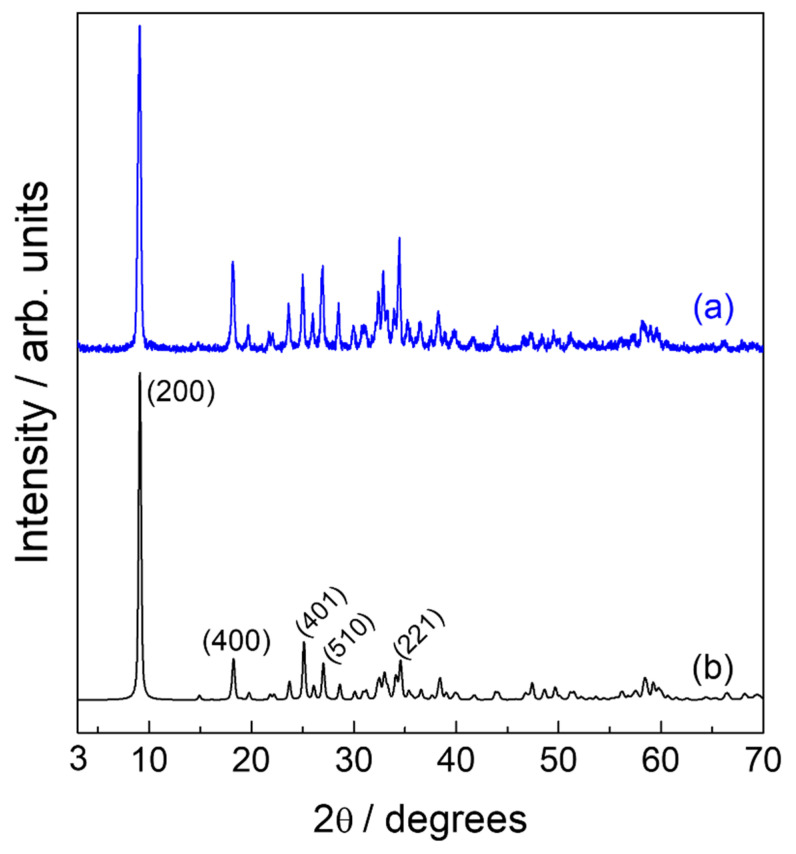
XRD pattern of the (**a**) Zn-NO_3_-LSH host matrix and (**b**) JCPDS-PDF n° 72-627 card.

Comparing the XRD pattern of the Zn-NO_3_-LSH host matrix with the JCPDS-PDF n° 72−627 card corresponding to zinc hydroxide nitrate (monoclinic unit cell and space group C2/m) and also comparing it to a series of LSH materials described in the literature [25,26,27,28,29,30,31], it is verified that the layered zinc hydroxide nitrate formation has regular stacking of sequential layers and phase purity (Figure A1). In the XRD pattern of the Zn-NO_3_-LSH, basal reflections (h00) situated in the low-angle region of 2θ are attributed to layer stacking, which is associated to basal distance [23,25]. The basal distance depends on size, geometry, conformation, and interlayer interactions of anionic species intercalated [25]. The other diffraction peaks of the layered zinc hydroxide nitrate are assigned to layered structures [32].

**Figure 1:**
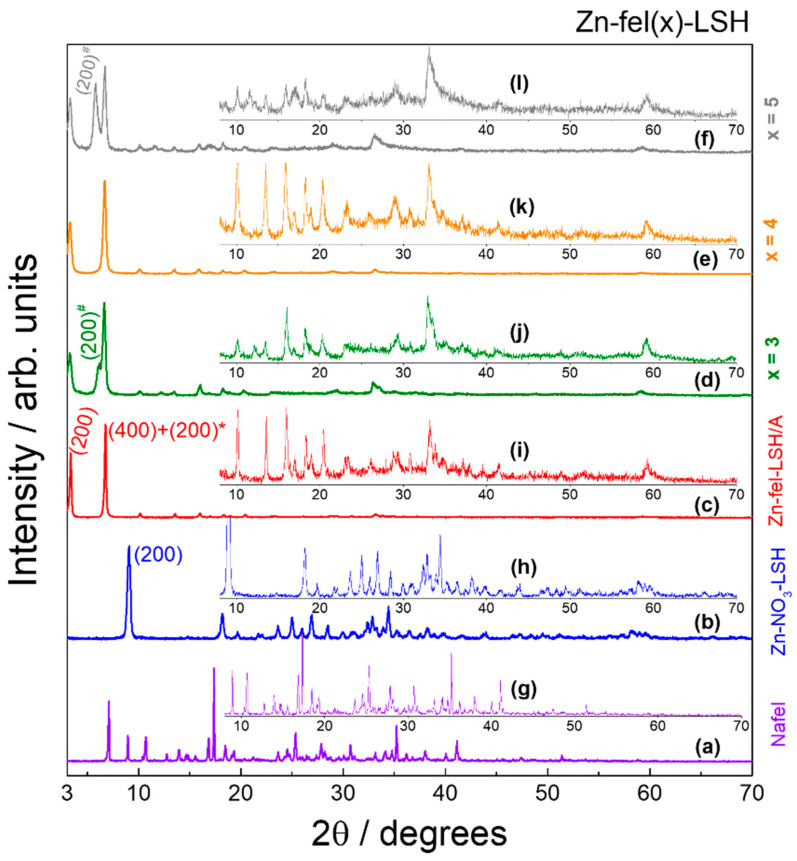
XRD patterns of (**a**) sodium ferulate salt (Nafel—NaC_10_H_9_O_4_), (**b**) Zn-NO_3_-LSH host matrix, (**c**) Zn-fel-LSH/A, (**d**) Zn-fel(3)-LSH, (**e**) Zn-fel(4)-LSH, and (**f**) Zn-fel(5)-LSH in the 3−70°/2θ region. Figures inserted show magnified X-ray diffraction lines of (**g**) Nafel salt (NaC_10_H_9_O_4_), (**h**) Zn-NO_3_-LSH host matrix, (**i**) Zn-fel-LSH/A, (**j**) Zn-fel(3)-LSH, (**k**) Zn-fel(4)-LSH, and (**l**) Zn-fel(5)-LSH in the 8−70°/2θ region.

**Figure 2:**
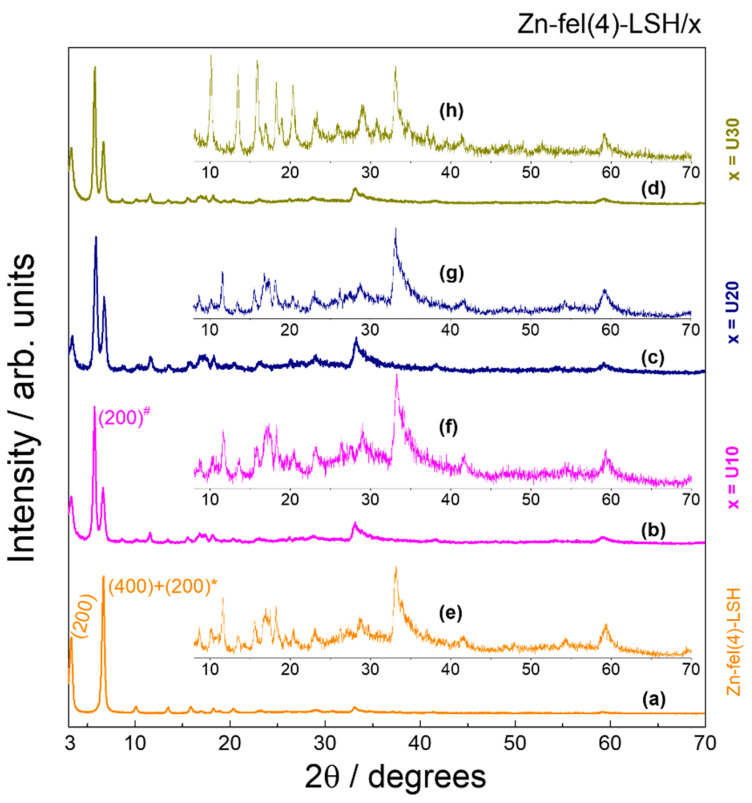
XRD patterns of (**a**) Zn-fel(4)-LSH, (**b**) Zn-fel(4)-LSH/U10, (**c**) Zn-fel(4)-LSH/U20, and (**d**) Zn-fel(4)-LSH/U30 in the 3−70°/2θ region. Figures inserted show magnified X-ray diffraction lines of (**e**) Zn-fel(4)-LSH, (**f**) Zn-fel(4)-LSH/U10, (**g**) Zn-fel(4)-LSH/U20, and (**h**) Zn-fel(4)-LSH/U30 in the 8−70°/2θ region.

The XRD patterns of LSH-ferulate materials (Figure 1 and Figure 2) show characteristic diffraction peaks of the Zn-NO_3_-LSH matrix, which indicate the formation of LSH materials. However, basal reflections of LSH-ferulate materials are shifted to smaller angles when compared with those of the host matrix. This displacement indicates the increase of the basal distance (Table 1) caused by intercalation of ferulate anions. Basal distance is calculated from the interplanar distance (d_hkl_) of respective basal reflections and interlayer spacing corresponds to difference between basal distance and zinc hydroxide nitrate sheet thickness [33]. Considering that the charge density of the layer and layered structure remain intact after the intercalation of anionic species [27] and assuming that there are only electrostatic and/or intermolecular interactions into interlayer galleries, the value of the zinc hydroxide nitrate sheet thickness corresponds to 10.00 Å [33].

Regardless of synthetic methods and/or the Zn^2+^/C_10_H_9_O_4_^−^ molar ratio used, LSH-ferulate samples are composed to a mixture of LSH phases that have different arrangements of ferulate anions in the interlayer region. In addition, the Zn^2+^/C_10_H_9_O_4_^−^ molar ratio is a synthesis parameter that influences the number of phases present in the Zn-fel(3)-LSH, Zn-fel(4)-LSH, and Zn-fel(5)-LSH materials; therefore, the Zn^2+^ and ferulate ions quantities are determinant to the formation of zinc hydroxide layers and interlayer arrangements of ferulate anions in these materials.

Ultrasound treatment is commonly used on layered materials to modify structural and morphological properties and/or to obtain ultrathin two-dimensional materials [34]. Considering the synthesis time and lowest number of LSH phases, the Zn-fel(4)-LSH material was subjected to ultrasound treatment for different times, giving rise to Zn-fel(4)-LSH/U10, Zn-fel(4)-LSH/U20, and Zn-fel(4)-LSH/U30 samples. In the XRD patterns of Zn-fel(4)-LSH/U10, Zn-fel(4)-LSH/U20, and Zn-fel(4)-LSH/U30 materials (Figure 2), it is observed the emergence of basal reflections associated to a new LSH phase present in the composition of these layered compounds. Therefore, cavitation effect produced by ultrasound treatment [35] provides the formation of new layered structures.

**Figure 3:**
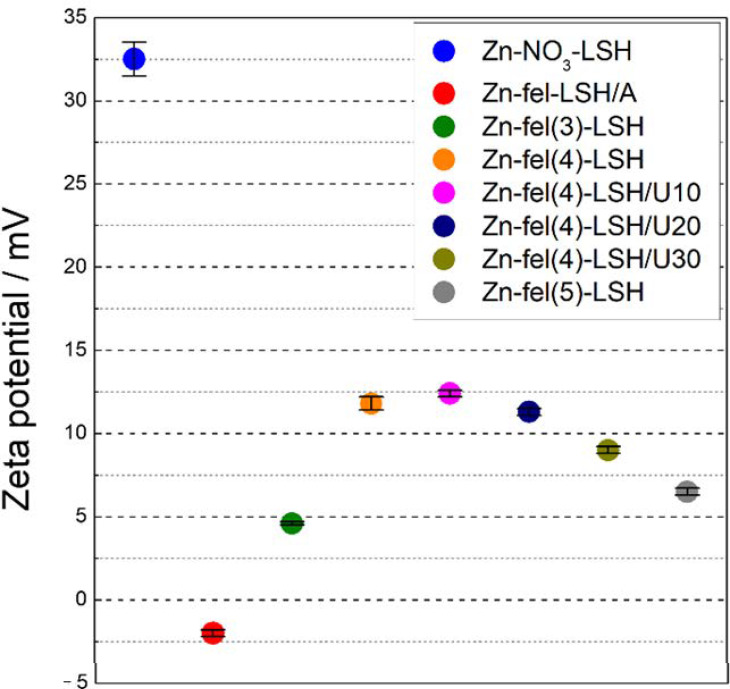
Zeta potential values of the Zn-NO_3_-LSH matrix and LSH-ferulate materials.

LSH-ferulate materials have lower zeta potential values than the Zn-NO_3_-LSH matrix (Figure 3) due to the interlayer and/or surface interactions between ferulate anions and LSH layers. This interfacial behavior is similar to those described for zinc layered hydroxide salts intercalated with organic anions [36,37]. The Zn-fel-LSH/A sample displays negative zeta potential, which indicates that the layered material has a negatively charged surface [38]. While, the other LSH-ferulate materials have a positively charged surface according to the zeta potentials obtained (Figure 3). The difference in the surface charge of the LSH-materials is correlated to the quantity of ferulate anions adsorbed in the zinc hydroxide layers, which influences the structure of the electrical double layer on LSH surfaces, i.e., Stern layer and diffusion layer [39]. The anions adsorbed thermally vibrate and can leave the Stern layer, consequently, there are always anions in the diffusion layer [40]. Thus, the charge of LSH particle associated to the Stern layer depends on concentration and surface interactions of ferulate anions. The negative charge surface of the Zn-fel-LSH/A sample comes from the greater amount of ferulate anions in the Stern layer that results in a smaller concentration of these anionic species in the diffuse layer. Therefore, the anion exchange method facilitates the adsorption of ferulate anions in the LSH host when compared to precipitation method. Moreover, zeta potentials of the LSH-ferulate materials depend on the Zn^2+^/C_10_H_9_O_4_^−^ molar ratio and ultrasound treatment time. Probably, constituent LSH phases of layered materials limit the amount of ferulate anions adsorbed, which provides different zeta potential values. 

**Figure 4:**
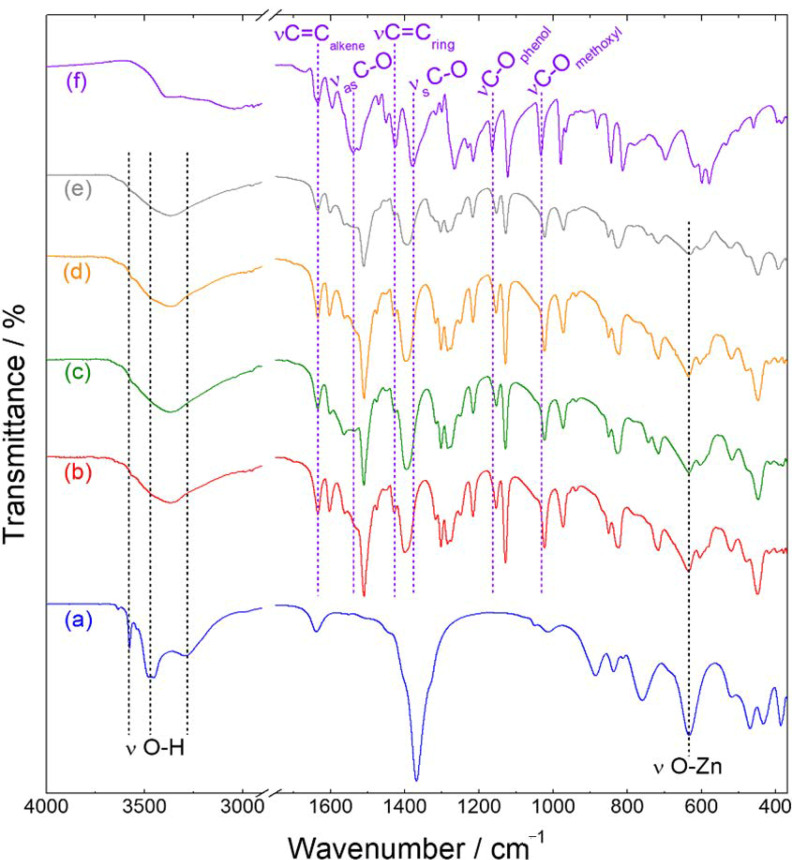
FTIR spectra of (**a**) Zn-NO_3_-LSH host matrix, (**b**) Zn-fel-LSH/A, (**c**) Zn-fel(3)-LSH, (**d**) Zn-fel(4)-LSH, (**e**) Zn-fel(5)-LSH, and (**f**) Nafel salt (NaC_10_H_9_O_4_).

The FTIR spectrum of the Zn-NO_3_-LSH matrix (Figure 4) shows a narrow band at 3576 cm^−1^ attributed to O-H stretching of hydroxyl anions belonging to the zinc hydroxide layers [25,41]. In addition, it is observed broad bands centered at 3468 and 3292 cm^−1^ assigned to hydroxyl stretching vibrations, which correspond to water molecules and hydroxyl groups linked with nitrate anions, respectively [41]. The vibrational spectrum of the host matrix also exhibits nitrate bands (ν_as_ = 1369 cm^−1^ and ν_s_ = 1015 cm^−1^) [42] and metal-oxygen vibrational modes (631, 521, 467, 430, and 386 cm^−1^) [41]. The FTIR spectrum of the sodium ferulate salt (NaC_10_H_9_O_4_) (Figure 4) shows characteristic bands of hydroxycinnamic acids, such as aromatic ring vibrations (1595, 1450, 1425, and 1215 cm^−1^) [43,44] and carboxylate stretching vibrations (ν_as_ = 1539 cm^−1^ and ν_s_ = 1379 cm^−1^) [45]. Moreover, the vibrational spectrum presents narrow bands at 1165 and 1032 cm^−1^ attributed to C-O stretching of the phenol and methoxy group, respectively [45].

**Figure 5:**
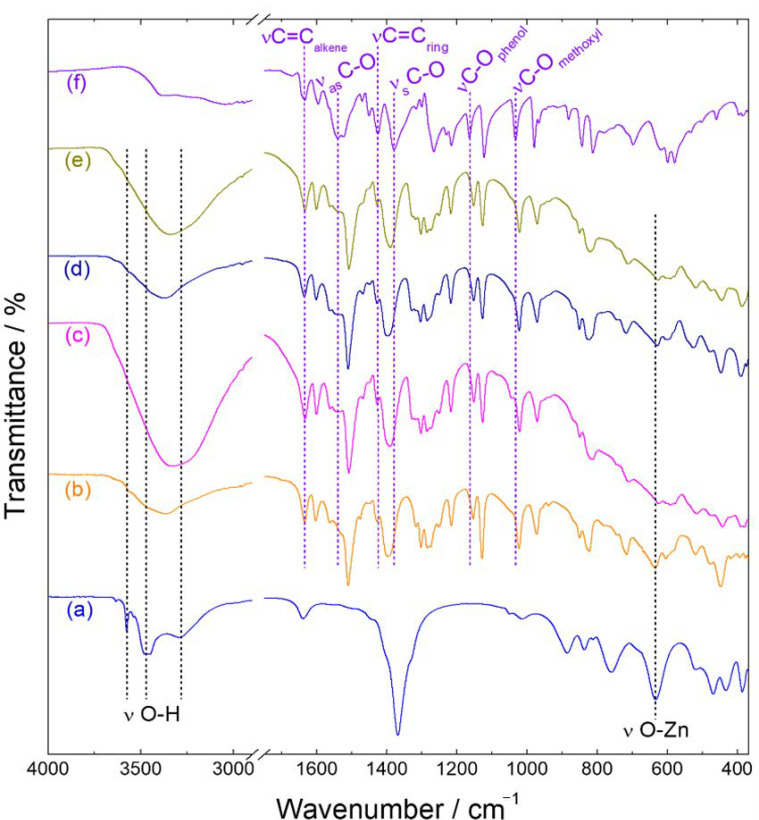
FTIR spectra of (**a**) Zn-NO_3_-LSH host matrix, (**b**) Zn-fel(4)-LSH, (**c**) Zn-fel(4)-LSH/U10, (**d**) Zn-fel(4)-LSH/U20, (**e**) Zn-fel(4)-LSH/U30, and (**f**) Nafel salt (NaC_10_H_9_O_4_).

In the FTIR spectra of LSH-ferulate materials (Figure 4 and Figure 5), characteristic bands of the ferulate anion are observed, such as C=C (alkene) stretching (1634 cm^−1^) and C=C (ring) stretching (1425 cm^−1^), and typical bands of the LSH host assigned to hydroxyl stretching vibrations and also the zinc-oxygen vibrational modes. Moreover, the phenol, methoxy, and carboxylate bands are shifted when compared to those of the ferulate anion. These vibrational shifts indicate that interactions between positive zinc hydroxide layers and ferulate anions occur through the polar groups of the guest anion. Host–guest interactions in these layered materials are also proven by enlargement and/or overlapping of hydroxyl bands and the displacement of metal-oxygen vibrational modes when compared to those of the Zn-NO_3_-LSH matrix. It is important to emphasize that methoxy and hydroxyl groups of ferulate anions interact with hydroxyl groups and water molecules of the LSH host by hydrogen bonds. 

Comparing the FTIR spectra of Zn-fel(4)-LSH/U10, Zn-fel(4)-LSH/U20, and Zn-fel(4)-LSH/U30 samples with the Zn-Fel(4)-LSH spectrum (Figure 5), it is observed the enlargement of the hydroxyl band of LSH samples subjected to ultrasound treatment caused by modifications of surface and/or interlayer interactions between ferulate anions, water molecules, and/or hydroxyl groups from layers. In addition, the increase of the relative intensity of the hydroxyl band in the Zn-fel(4)-LSH/U10 and Zn-fel(4)-LSH/U30 materials can be associated to a greater amount of water molecules adsorbed and/or intercalated. Therefore, ultrasound cavitation that induces the break and/or the formation of different host–guest interactions in these layered materials is a function of the treatment time.

The carboxylate groups of ferulate anions can be coordinated to zinc ions in different ways; therefore, the frequency difference between carboxylate asymmetric and symmetric stretching vibrations (Δν = ν_as_ − ν_s_) gives information about the coordination mode [42] having the Δν value of the sodium ferulate salt as reference. Thus, the monodentate coordination mode is found for Δν values much greater than the reference salt due to the frequency increase of the carboxylate asymmetric stretching vibration and the frequency decrease of the symmetric stretching vibration when compared to those of the ionic compound [46]. The chelate-bidentate mode corresponds to Δν values significantly lower than the reference salt because the frequency of the carboxylate asymmetric stretching vibration decreases and the frequency of the carboxylate symmetric stretching vibration increases [46]. The Δν values for the bridging bidentate mode are close to the sodium ferulate salt value [42,46].

According to the Δν values obtained for LSH-ferulate materials (Table 2), carboxylate groups are bound to the Zn^2+^ ions from the layers via bridging bidentate and/or chelate-bidentate mode. This result indicates that there are different interaction modes between ferulate anions and LSH layers, which can be associated to several arrangements of ferulate species in the interlayer spacing of layered materials. In addition, each LSH-ferulate sample has carboxylate groups coordinated to metal ions in two different ways. Therefore, constituent LSH phases of layered materials confirmed in XRD results (Figure 1 and Figure 2) are directly related to coordination modes of the carboxylate groups assigned to ferulate anions. Thus, the coordination of guest anions causes zinc hydroxide sheet thickness changes, which explains the small interlayer spacing values situated in the 3–5 Å range (Table 1).

In the Zn-fel-LSH/A material, the Δν value equal to the Nafel salt (NaC_10_H_9_O_4_) indicates an ionic mode of carboxylate groups attributed to ferulate anions. These ionic interactions of carboxylate groups can be associated to an excess amount of ferulate anions in the surface of the layered material as seen in the zeta potential results.

**Figure 6:**
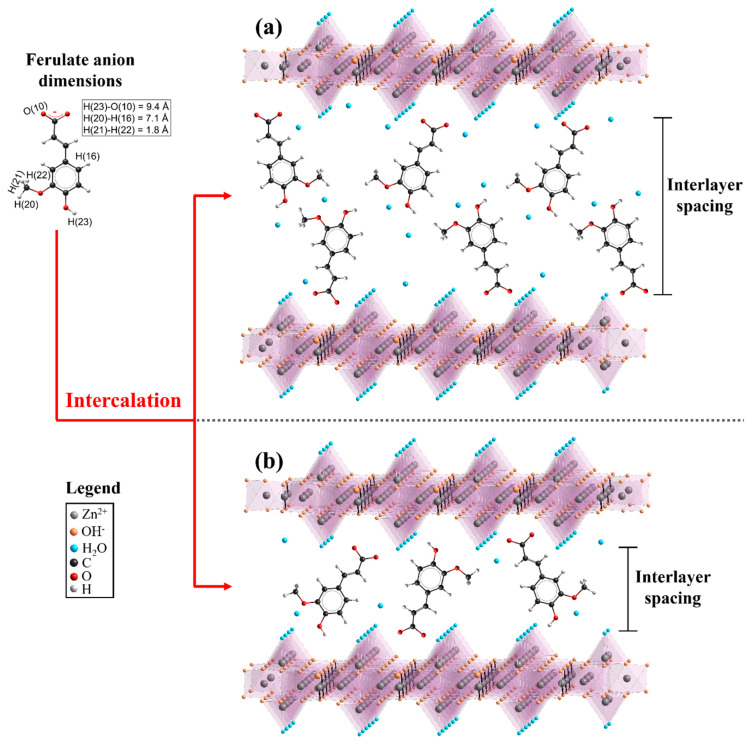
Schematic representations of proposed (**a**) bilayer and (**b**) monolayer arrangements for the ferulate anions in the LSH-ferulate materials.

Considering the ferulate anion dimensions achieved by semi-empirical calculations (1.8 × 7.1 × 9.4 Å), interlayer distances of LSH-ferulate materials from XRD data (Table 1) and host–guest interactions described in FTIR results, mono and/or bilayer arrangements of organic anions in the interlayer region are proposed. In the bilayer arrangements (Figure 6a), carboxylate groups of ferulate anions are close to the positive LSH layers and hydroxyl and methoxy groups are interacting with each other and/or water molecules in the interlayer spacing. The monolayer arrangements (Figure 6b) are formed by interactions between polar groups of ferulate anions and positively charge layers, which cause probably molecular structure distortions of the guest anion. Therefore, the interlayer spacing values of monolayer arrangements are associated with spatial orientations of ferulate species in the interlayer galleries.

**Figure 7:**
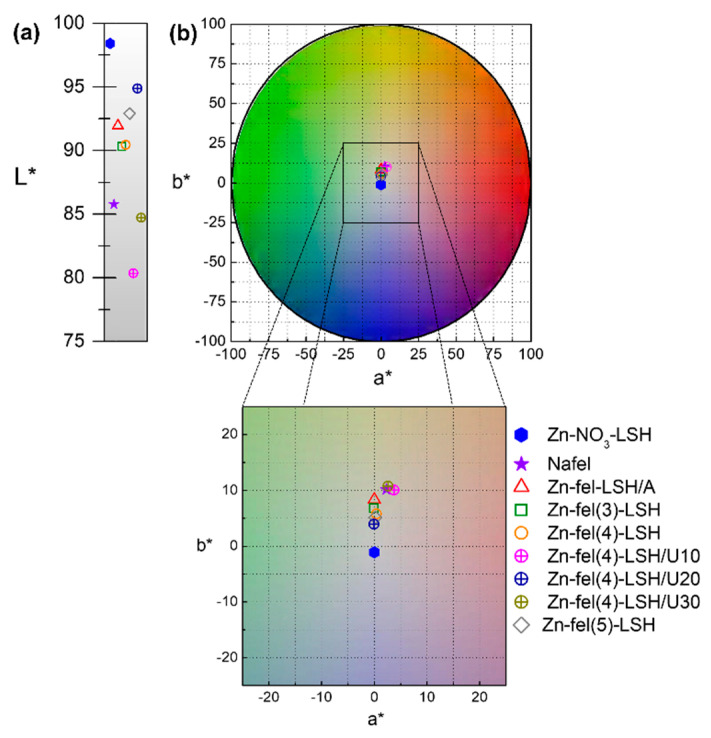
(**a**) Brightness scale; (**b**) Color scale corresponding to the CIELab color diagram of Zn-NO_3_-LSH matrix, Nafel salt (NaC_10_H_9_O_4_), and LSH-ferulate materials.

**Figure A2:**
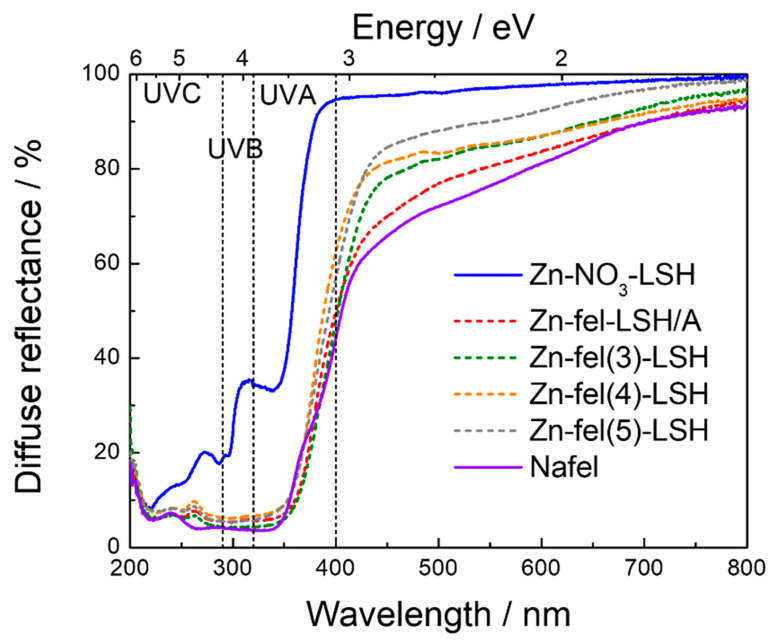
Diffuse reflectance spectra of Zn-NO_3_-LSH, Zn-fel-LSH/A, Zn-fel(3)-LSH, Zn-fel(4)-LSH, Zn-fel(5)-LSH and Nafel (NaC_10_H_9_O_4_) salt.

**Figure A3:**
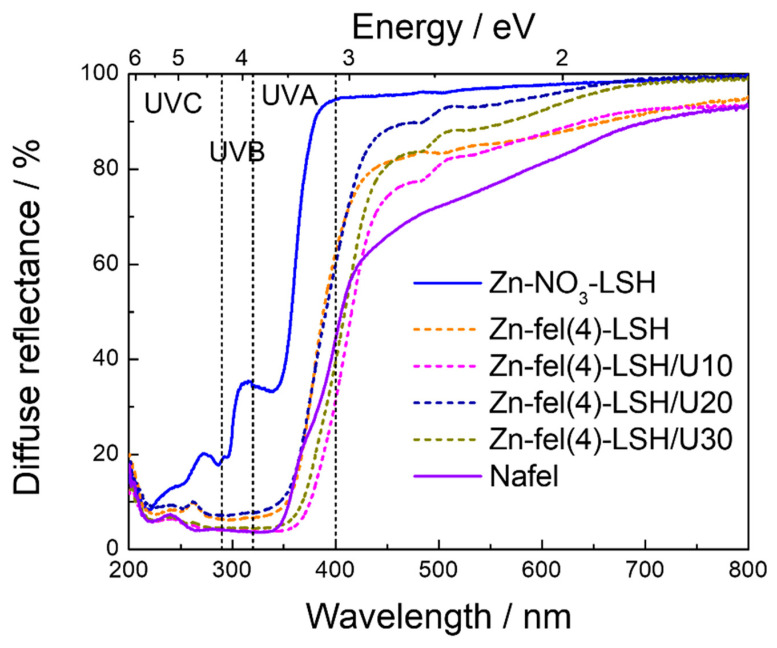
Diffuse reflectance spectra of Zn-NO_3_-LSH, Zn-fel(4)-LSH, Zn-fel(4)-LSH/U10, Zn-fel(4)-LSH/U20, Zn-fel(4)-LSH/U30 and Nafel (NaC_10_H_9_O_4_) salt.

The CIELab color diagram (Figure 7) shows that LSH-ferulate materials have a yellowish-white color; consequently, their use in sunscreen formulations do not compromise the desired aesthetics appearance for the cosmetic products. In the UV-VIS diffuse reflectance spectra (Figure A2 and Figure A3), it is observed that LSH-ferulate materials have absorption edge situated in the 350–400 nm region similar to Nafel salt. Moreover, each layered material has a characteristic visible-light scattering (400–800 nm) due its particle size and refractive index associated to constituent LSH structures.

**Figure 8:**
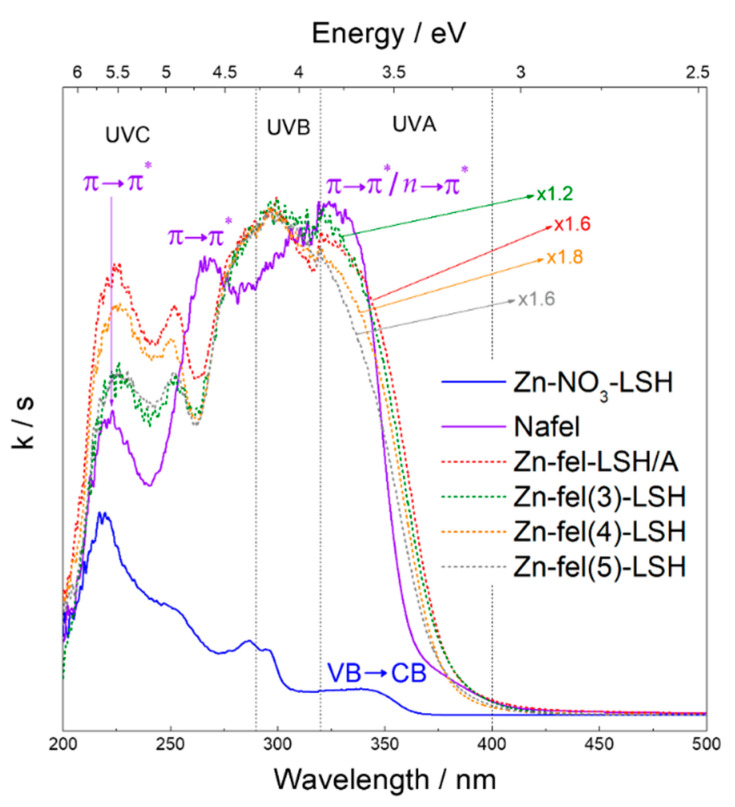
Absorption spectra of Zn-NO_3_-LSH, Zn-fel-LSH/A, Zn-fel(3)-LSH, Zn-fel(4)-LSH, Zn-fel(5)-LSH, and Nafel salt (NaC_10_H_9_O_4_).

The absorption spectrum of the Zn-NO_3_-LSH matrix (Figure 8) shows broad bands with maximum values at 220, 251, 287, and 296 nm, which are assigned to electronic transitions of the nitrate anions intercalated [47,48]. This spectrum also exhibits an absorption band at 345 nm attributed to VB→CB transitions of the zinc hydroxide layers. The Nafel spectrum (Figure 8) presents characteristic absorption bands of hydroxycinnamic compounds situated in the 210–280 nm region, which are assigned to π→π* electronic transitions [49,50]. In addition, a broad and intense band at 325 nm attributed to a mixture of π→π* and *n*→π* transitions [49,50] is observed. These last electronic transitions correspond to carboxyl group and π-aromatic system of the ferulate anion.

**Figure 9:**
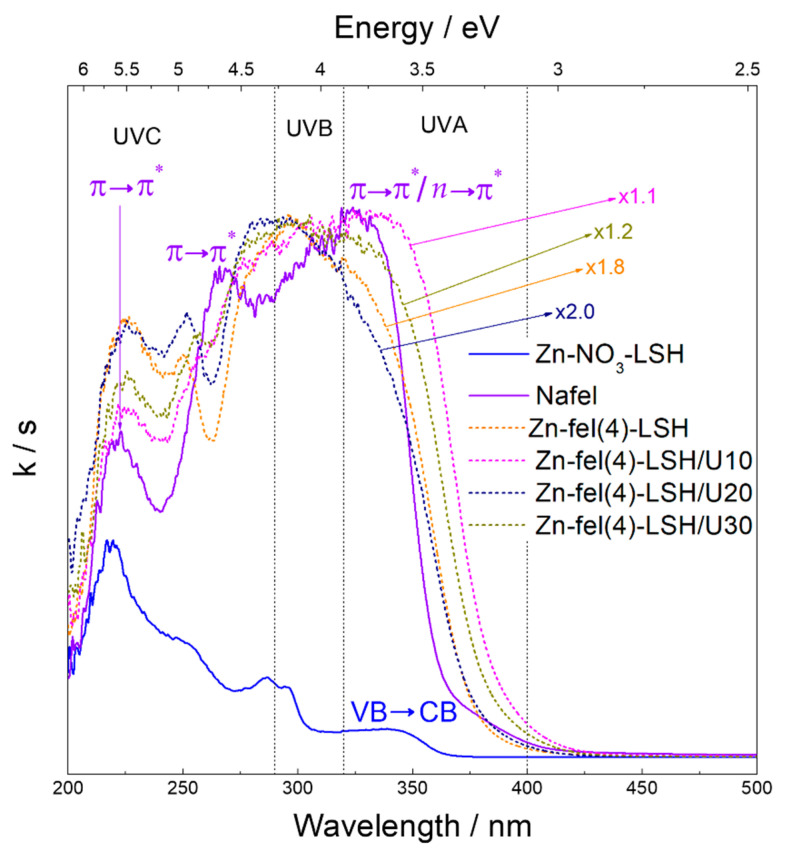
Absorption spectra of Zn-NO_3_-LSH, Zn-fel(4)-LSH, Zn-fel(4)-LSH/U10, Zn-fel(4)-LSH/U20, Zn-fel(4)-LSH/U30, and Nafel salt (NaC_10_H_9_O_4_).

Absorption spectra of LSH-ferulate materials (Figure 8 and Figure 9) show typical absorption bands of host matrix and ferulate anion, although these bands are shifted, enlarged, and/or overlapped when compared to those of Zn-NO_3_-LSH matrix and Nafel salt. The absorption band situated in the 240–260 nm region corresponds to an overlapping of the nitrate band of the LSH host and hydroxycinnamic band assigned to π→π* transitions of ferulate anions. This absorption band indicates the presence of nitrate anions in the LSH-ferulate materials, which are co-intercalated with ferulate anions in the interlayer galleries. Moreover, host–guest interactions in these layered materials provide the hydroxycinnamic band shifts due to changes in the π-aromatic system of ferulate anions. The enlarged absorption band in the 270–380 nm region is composed to the mixture of the LSH band attributed to VB→CB transitions and ferulate band assigned to a mixture of π→π* and *n*→π* transitions. The bathochromic shift of this band in the LSH-ferulate materials indicates that carboxylate groups are coordinated to the Zn^2+^ ions from the host layers. The bathochromic effect is associated to energy decrease of π→π* transitions of carboxylate groups generally assigned to the energy stabilization of the π-system [50]. The five-membered ring structures can stabilize π-systems. According to FTIR results, ferulate anions are bound via bridging bidentate and/or chelate-bidentate mode, consequently, bridging bidentate mode can be suggested. The Zn-fel(4)-LSH/U10 and Zn-fel(4)-LSH/U30 materials have different absorption profiles than other LSH-ferulate materials. This difference provides evidence that ultrasonic cavitation also causes energy levels changes of the LSH-ferulate system due to modifications of surface and/or interlayer interactions between ferulate anions and host layers proven in the FTIR results. The UV absorption capacity of LSH-ferulate materials in the 210–380 nm region demonstrates their potential for applicability as active constituents of photoprotective products.

**Figure 10:**
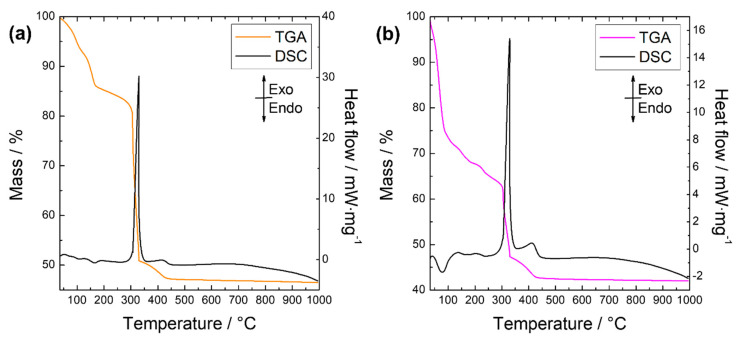
TGA-DSC curves of (**a**) Zn-fel(4)-LSH and (**b**) Zn-fel(4)-LSH/U10 samples.

Considering the shortest synthesis time, ultrasound treatment effects, structural properties, and UV shielding ability, Zn-fel(4)-LSH and Zn-fel(4)-LSH/U10 materials were chosen as reference samples to investigate thermal decomposition behavior, antioxidant activity, and sun protection factor (SPF) performance of LSH intercalated with ferulate anions. TGA-DSC curves of Zn-fel(4)-LSH and Zn-fel(4)-LSH/U10 samples (Figure 10) show four main thermal events. The first two events associated to endothermic processes occur until 300 °C and correspond to loss of water molecules adsorbed and intercalated in the layered materials [25,26]. The further thermal events (exothermic processes) occur in the 300–450 °C temperature range and are attributed to the simultaneous decomposition of guest species (ferulate combustion) and LSH layers (layers dehydroxylation) [25,26].

Although the layered materials exhibit similar thermal decomposition profiles, the percentages of mass loss (wt%) corresponding to main thermal events are different (Table 3). This difference in the chemical composition comes from the distinct LSH phases that compose the Zn-fel(4)-LSH and Zn-fel(4)-LSH/U10 materials as noted in the XRD results. In addition, the Zn-fel(4)-LSH/U10 sample has a smaller amount of ferulate anions than the Zn-fel(4)-LSH material. However, the Zn-fel(4)-LSH/U10 material presents a high amount of water molecules in its chemical composition, which can be correlated to the relative intensity of the hydroxyl band observed in FTIR results. Again, it is verified that ultrasonic cavitation induces specific interactions between ferulate species, water molecules, and LSH layers, influencing on the quantity of water and organic anions adsorbed and/or intercalated.

**Figure A4:**
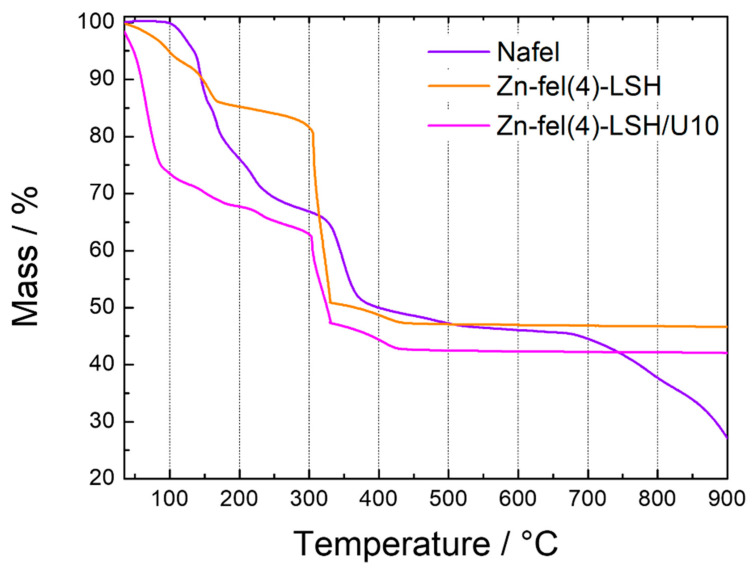
TGA curves of Nafel salt (NaC_10_H_9_O_4_), Zn-fel(4)-LSH and Zn-fel(4)-LSH/U10 samples.

The TGA curve of the Nafel salt (Figure A4) shows that thermal decomposition of this organic compound occurs in four main steps. The decomposition steps are observed in the following temperature range (wt %): 100–315 °C (34%), 315–395 °C (16%), 395–677 °C (5.0%), and 677–900 °C (18%). The first step is related to water loss and partial decomposition of the sodium ferulate salt. Analyzing the temperature range of the initial decomposition of ferulate anions in the layered materials and Nafel salt, it is noted that the intercalation process provides the increase of the thermal stability of ferulate species. This increase of the thermal stability is associated to the confinement of ferulate anions in the interlayer region, which changes the oxidation mechanisms of organic species. Therefore, great thermal stability of intercalated ferulate anions is an advantageous aspect for use of the LSH-ferulate materials in photoprotective products.

**Figure A5:**
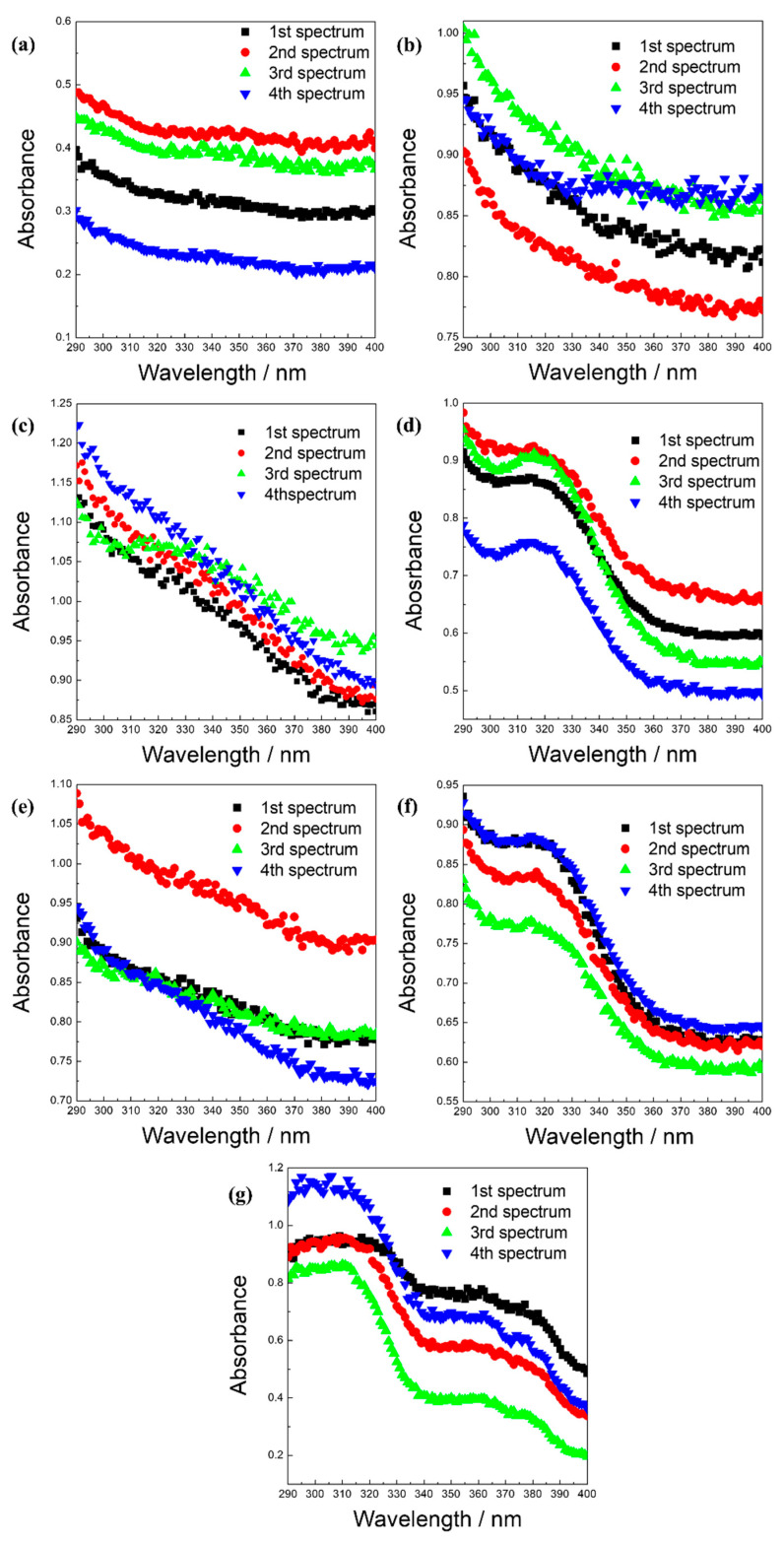
Absorption spectra measurements of (**a**) Base cream, (**b**) Zn-NO_3_-LSH cream, (**c**) Zn-fel(4)-LSH cream, (**d**) Nafel/2 cream, (**e)** Zn-fel(4)-LSH/U10 cream, (**f**) Nafel/1 cream and (**g**) commercial sunscreen films.

The UV shielding performance of cosmetic formulations containing Zn-fel(4)-LSH and Zn-fel(4)-LSH/U10 materials as active ingredients of the photoprotective products was analyzed by the *in vitro* sun protection factor (SPF) method. The *in vitro* SPF method is based on UV-VIS spectrophotometric measurements [51,52]; consequently, absorption spectra measurements of photoprotective creams films were realized (Figure A5) to obtain *in vitro* SPF values. A commercial sunscreen product (SPF labeled equal to 10) was used as a reference standard for assessing the reliability and consistency of the SPF results. Comparing the SPF labeled (SPF equal to 10) and the SPF experimental of the commercial sunscreen (Table 4), it is verified that the SPF values are close, indicating that the *in vitro* method used allows the evaluation of the UV shielding capacity of cosmetic formulations.

In order to facilitate the identification of the cosmetic formulations, the skin care formulation without active ingredients was designated base cream and other formulations were named with the same acronyms of samples dispersed in the colloidal system. So, the formulation containing zinc hydroxide nitrate host matrix was named Zn-NO_3_-LSH cream and formulations containing LSH-ferulate materials were designated Zn-fel(4)-LSH and Zn-fel(4)-LSH/U10 creams. In addition, cosmetic formulations containing 0.1% and 1.8 wt% of the sodium ferulate salt were named Nafel/1 and Nafel/2 creams, respectively. To compare UV shielding ability of the LSH-ferulate materials and ferulate species isolated, Nafel/1 and Nafel/2 cream formulations have the same amount of ferulate anions present in the chemical composition of Zn-fel(4)-LSH/U10 and Zn-fel(4)-LSH samples, respectively.

The Zn-NO_3_-LSH, Zn-fel(4)-LSH/U10, Nafel/1, Zn-fel(4)-LSH, and Nafel/2 creams have higher SPF values than the base cream (Table 4) due to the colloidal dispersion of ferulate molecules or layered materials (Zn-NO_3_-LSH and LSH-ferulate samples) in the cosmetic formulations. These compounds dispersed in the skin care cream have UV absorption capacity as seen in the UV-VIS absorption spectra (Figure 8 and Figure 9). Based on the literature [53,54], sunscreens that have SPF ≤ 15 prevent damages to human skin caused by excessive exposure to UVB radiation; therefore, cosmetic formulations obtained present UVB protection.

The SPF values of Zn-NO_3_-LSH, Zn-fel(4)-LSH/U10, Nafel/1, and Nafel/2 creams are close to each other (Table 4), indicating a similar UV protection performance. However, higher SPF value of the Zn-fel(4)-LSH cream shows a better UV shielding performance, which demonstrates the potential of the Zn-fel(4)-LSH material as active constituent of sunscreens. Therefore, synergistic effects from host–guest interactions between ferulate anions and LSH layers are responsible for high UV shielding ability of the Zn-fel(4)-LSH material.

The smaller UV shielding capacity of the Zn-fel(4)-LSH/U10 cream when compared to the Zn-fel(4)-LSH formulation can be related to the amount of ferulate anions present in the chemical composition and/or optical properties of the colloidal system resulting from intermolecular interactions between layered material particles and formulation constituents. Ultrasound cavitation causes surface modifications of the Zn-fel(4)-LSH/U10 particles observed by zeta potential measurements (Figure 3) affecting the interfacial structuring in the colloidal system.

**Figure A6:**
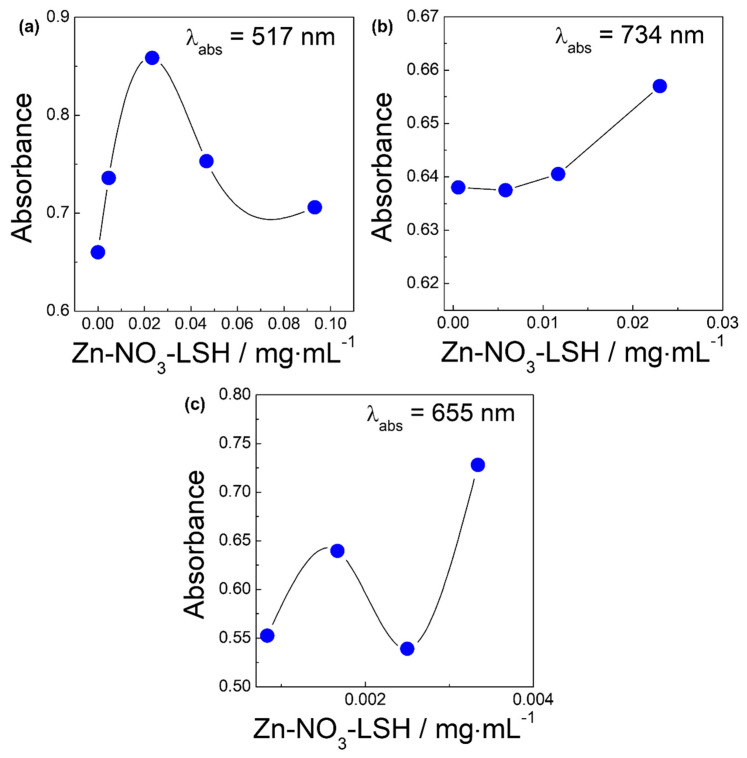
Average absorbance of (**a**) DPPH^•^ and (**b**)ABTS^•+^ reactive species as function of Zn-NO_3_-LSH concentration in the DPPH^•^ and ABTS^•+^ assay, respectively. Average absorbance of the (**c**) TMB chromophore in presence of HOCl/OCl^−^ species as function of host matrix concentration.

**Figure A7:**
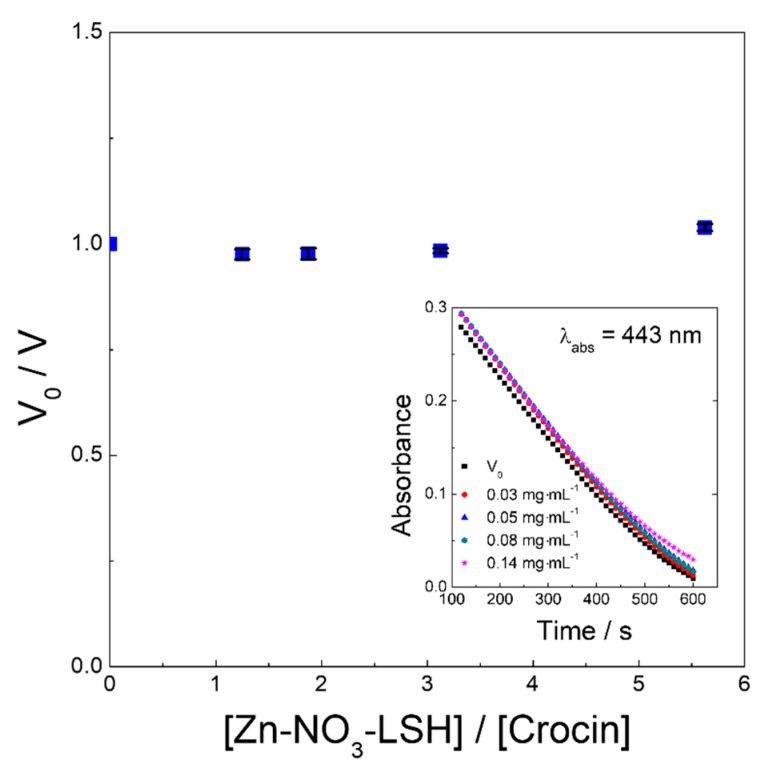
Representation between velocities and concentrations of the Zn-NO_3_-LSH matrix in the crocin bleaching assay. V_0_, velocity in the absence of the host matrix; V, velocity in the presence of the Zn-NO_3_-LSH matrix.

High concentrations of reactive oxygen species (ROS) combined to deficit of cellular defense mechanisms cause damages to the human organism [55], e.g., lipoperoxidation [56]. Thus, ROS effects can be minimized by antioxidant compounds [57]. In this perspective, materials that have UV shielding ability and antioxidant activity perform simultaneously beneficial functions to the human organism; consequently, they can be denominated multifunctional filters [58,59]. The antioxidant capacity of the Zn-NO_3_-LSH, LSH-ferulate materials, and Nafel was investigated by spectrophotometric methods [57] using model radicals (DPPH^•^ and ABTS^•+^) and radical (ROO^•^) and non-radical (HOCl) reactive species of occurrence in biological systems. It is important to mention that the Zn-NO_3_-LSH host matrix does not present antioxidant activity against to reactive species investigated in this work (Figure A6 and Figure A7).

The DPPH^•^ is a free radical that has an unpaired electron in the nitrogen atom that is conjugated to the aromatic ring [60]. This reactive species shows a UV-VIS absorption band with maximum value at 517 nm [60]. Redox reactions between DPPH^•^ radicals and antioxidant compounds cause the reduction of DPPH^•^ species, providing the intensity decrease of absorption band at 517 nm. Thus, antioxidant capacity of analyzed compounds is directly related to intensity of this characteristic absorption band of DPPH^•^. The linear relationship between concentration of the antioxidant compound and capture percentage of DPPH^•^ species allows to calculate the effective concentration (EC_50_) of the antioxidant sample needed to capture 50% of the radicals present in the solution through the equation obtained by linear regression. The EC_50_ parameter in percentage is ordinarily used to compare the antioxidant activity of different chemical compounds [57].

**Figure 11:**
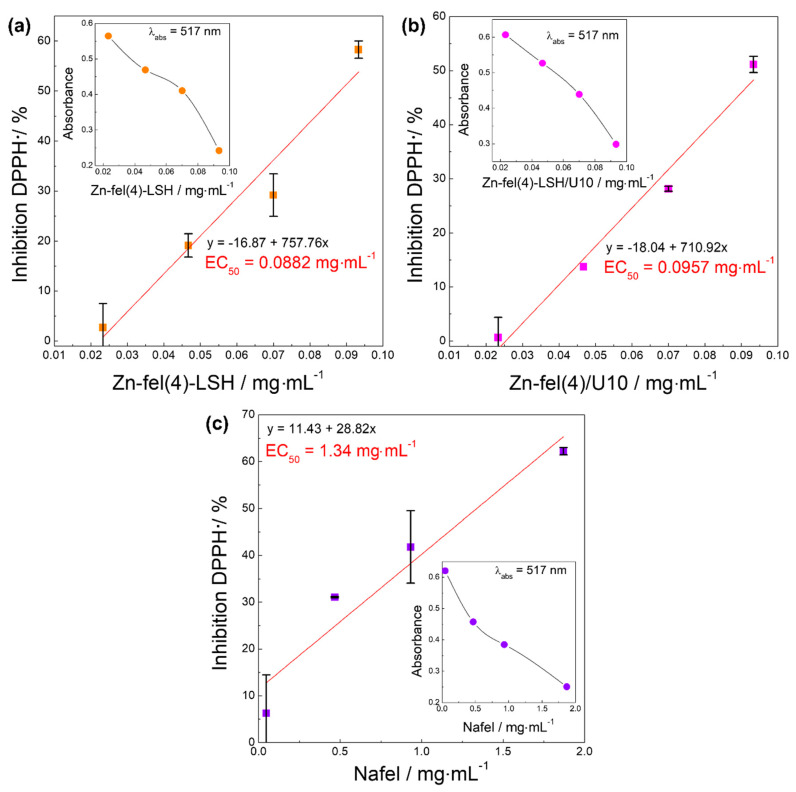
Inhibition (capture) of DPPH^•^ radicals by (**a**) Zn-fel(4)-LSH, (**b**) Zn-fel(4)-LSH/U10, and (**c**) Nafel (NaC_10_H_9_O_4_) samples. Figures inserted show average absorbance of DPPH^•^ species as function of the sample concentration.

Analyzing the results of the DPPH assay (Figure 11), it is verified that Nafel salt and LSH-ferulate materials have DPPH^•^ capturing ability due to the increase of the percentage capture of these reactive species with the concentration increase of these antioxidant compounds. The EC_50_ values obtained for Zn-fel(4)-LSH, Zn-fel(4)-LSH/U10, and Nafel compounds are 0.0882, 0.0957, and 1.34 mg mL^−1^, respectively. Therefore, the following increasing order in DPPH^•^ capture efficiency is observed: Nafel < Zn-fel(4)-LSH/U10 < Zn-fel(4)-LSH. This DPPH^•^ capture efficiency of LSH-ferulate materials indicates their potential as multifunctional filters.

**Figure 12:**
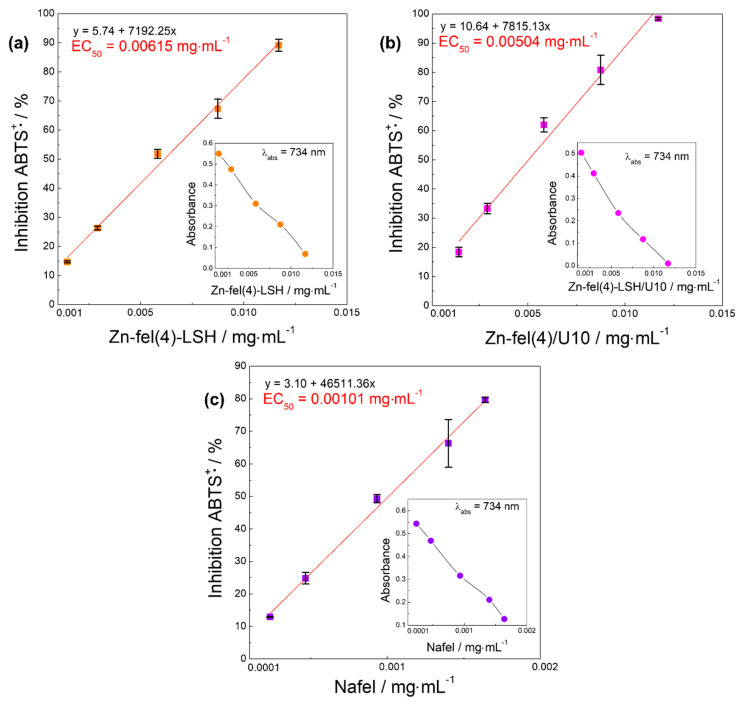
Inhibition (capture) of ABTS^•+^ radicals by (**a**) Zn-fel(4)-LSH, (**b**) Zn-fel(4)-LSH/U10, and (**c**) Nafel (NaC_10_H_9_O_4_) samples. Figures inserted show average absorbance of ABTS^•+^ species as function of the sample concentration.

Similar to the DPPH^•^ assay, the method of ABTS^•+^ cation capture is based on oxidation-reduction reactions analyzed by UV-VIS spectrophotometric measurements [57]. In this method, the intensity decrease of the absorption band of ABTS^•+^ species at 734 nm is proportional to the antioxidant concentration increase [61]. Again, the EC_50_ parameter is used to express antioxidant capacity. The results of the ABTS^•+^ test (Figure 12) show that Nafel salt and LSH-ferulate materials present ABTS^•+^ scavenging activity. The EC_50_ values of Zn-fel(4)-LSH, Zn-fel(4)-LSH/U10, and Nafel samples are 0.00615, 0.00504, and 0.00101 mg mL^-1^, respectively; thus, the increasing order in efficiency for capturing the ABTS^•+^ species is Zn-fel(4)-LSH < Zn-fel(4)-LSH/U10 < Nafel, the inverse capture efficiency order of the DPPH^•^ assay.

**Figure 13:**
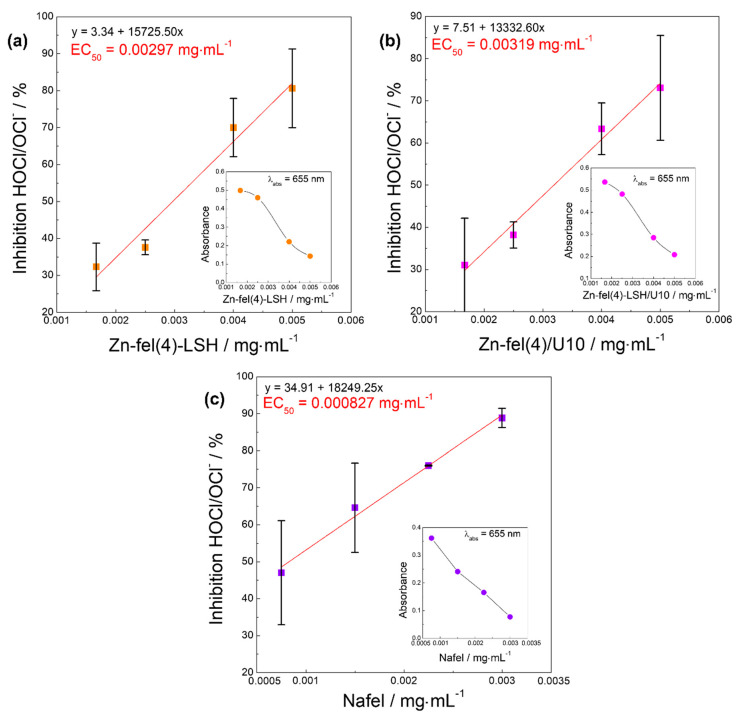
Inhibition (capture) of HOCl/OCl^−^ reactive species by (**a**) Zn-fel(4)-LSH, (**b**) Zn-fel(4)-LSH/U10 and (**c**) Nafel (NaC_10_H_9_O_4_) samples. Figures inserted show average absorbance of the TMB chromophore in presence of HOCl/OCl^−^ species as function of sample concentration.

Hypochlorous acid (HOCl) is a non-radical reactive species, which has strong antimicrobial activity in the human organism [62]. However, high reactivity of the HOCl combined to propensity to permeate membranes can oxidize biomolecules causing cellular damages [62]. In the hypochlorous acid scavenging assay, the antioxidant compound interacts with HOCl/OCl^−^ species and prevents the formation of the blue chromophore compound produced by oxidation of the TMB [63]. Thus, the intensity decrease of the absorption band of blue chromophore at 655 nm is proportional to the increase of the antioxidant concentration. Based on the results of this assay (Figure 13), it is noted that LSH-ferulate materials and Nafel salt have HOCl/OCl^−^ inhibiting capacity and the increasing order in efficiency for capturing HOCl/OCl^−^ species is Zn-fel(4)-LSH/U10 (EC_50_ = 0.00319 mg mL^−1^) < Zn-fel(4)-LSH (EC_50_ = 0.00297 mg mL^−1^) < Nafel (EC_50_ = 0.000827 mg mL^−1^). Therefore, LSH-ferulate materials and Nafel salt act as HOCl/OCl^−^ sequestrants and can reduce tissue damages caused by attacks of microorganisms and inflammatory processes [57].

**Figure 14:**
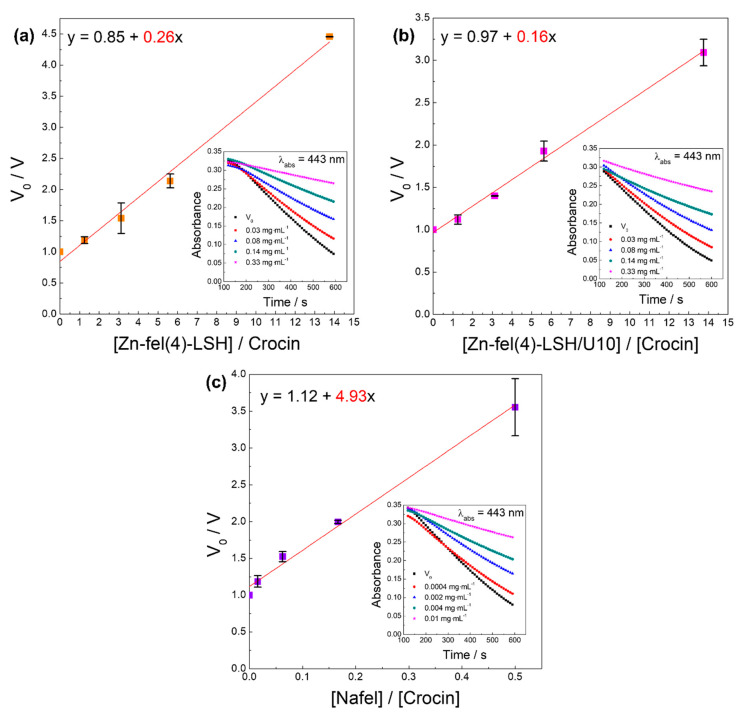
Representation between velocities and concentrations of (**a**) Zn-fel(4)-LSH, (**b**) Zn-fel(4)-LSH/U10, and (**c**) Nafel (NaC_10_H_9_O_4_) samples in the crocin bleaching assay. V_0_, velocity in the absence of LSH-ferulate materials and Nafel salt; V, velocity in the presence of LSH-ferulate materials and Nafel salt.

**Figure A8:**
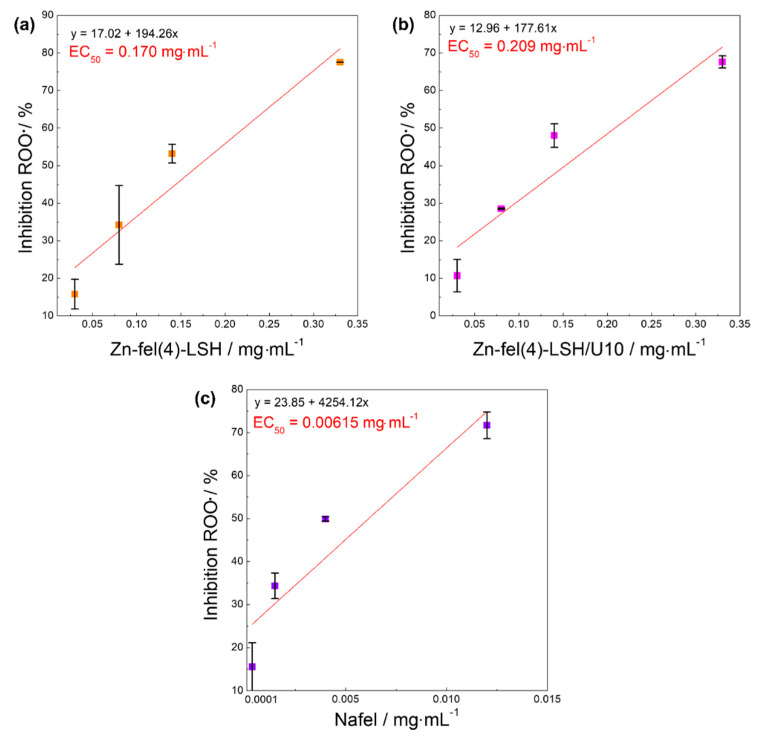
Inhibition (capture) of ROO^•^ reactive species by (**a**) Zn-fel(4)-LSH, (**b**) Zn-fel(4)-LSH/U10 and (**c**) Nafel (NaC_10_H_9_O_4_) samples.

The ROO^•^ is a radical reactive species and is produced by the lipoperoxidation process (LPO) [63]. Among experimental methods used to investigate the inhibition of the LPO mechanism, Crocin bleaching assay is suitable to evaluate the antioxidant activity against ROO^•^ radicals [57,63]. This assay is based on the bleaching rate of the crocin solution in the presence of antioxidants; therefore, the ROO^•^ capturing ability depends on kinetic competition between crocin and antioxidant compounds. The ROO^•^ scavenging activity is directly related to the angular coefficient obtained by linear regression from V_0_/V *versus* [Antioxidant]/[Crocin] graphs. Higher angular coefficient values indicate higher antioxidant activity of samples. The results of the Crocin bleaching assay (Figure 14 and Figure A8) show that samples exhibit ROO^•^ scavenging activity; therefore, LSH-ferulate materials have potential to reduce the LPO of cellular membranes. According to slope and EC_50_ values of samples (Table 5), the increasing order of the antioxidant ability is: Zn-fel(4)-LSH/U10 < Zn-fel(4)-LSH < Nafel.

The reactive species assays realized show that Zn-fel(4)-LSH and Zn-fel(4)-LSH/U10 materials have capacity to capture DPPH^•^, ABTS^•+^, ROO^•^, and HOCl/OCl^−^ reactive species. This antioxidant behavior indicates that LSH-ferulate materials cause decrease and/or inhibition of reactive species probably through to redox reactions with ferulate anions present in the chemical composition of these layered materials and/or interactions between LSH layers and reactive species. The Zn-fel(4)-LSH material exhibits a better DPPH^•^, ROO^•^, and HOCl/OCl^−^ scavenging activity than the Zn-fel(4)-LSH/U10 sample, which can be related to a greater amount of ferulate anions in its composition as seen in TGA/DSC results (Table 3). In the ABTS^•+^ assay, inverse order in efficiency for capturing the reactive species can be correlated to unpaired electron distribution in the π-system and/or positive charge of the ABTS^•+^ radicals. Therefore, antioxidant and UV shielding abilities exhibited by LSH-ferulate materials confirm their potential as multifunctional filters, mainly the Zn-fel(4)-LSH material.

## 3. Materials and Methods

### 3.1. Materials

For the synthesis of layered materials, zinc nitrate hexahydrate (Zn(NO_3_)_2_.6H_2_O—99.98%, Synth, Diadema, Brazil), ferulic acid (C_10_H_10_O_4_—99.99%, Sigma-Aldrich, St. Louis, MO, USA), and sodium hydroxide (NaOH—98.67%, Synth) were used without further purification. The sodium ferulate salt (NaC_10_H_9_O_4_) was obtained by mixing C_10_H_10_O_4_ and NaOH, at a 1:1 molar ratio, in a mixed solution of ethanol and water. In the preparation of cosmetic formulations, dipropan-2-yl hexanedioate (Dhaymers, Taboão da Serra, Brazil), 2,3-dihydroxypropyl octadecanoate (Via Farma, São Paulo, Brazil), mixture of hexadecan-1-ol and octadecan-1-ol (Cetostearyl alcohol, Via Farma), mixture of hexadecan-1-ol, octadecan-1-ol, and oxirane (Cosmowax^®^ J, Croda, Campinas, Brazil), (1-decanoyloxy-3-octanoyloxypropan-2-yl) dodecanoate (Via Farma), propane-1,2-diol (Qhemis, Jundiaí, Brazil), methyl 4-hydroxybenzoate (Synth), propyl 4-hydroxybenzoate (Synth), 2,2′,2″,2‴-(1,2-Ethanediyldinitrilo)tetraacetic acid (Qhemis) were used without further purification. The chemical compounds used in the reactive species scavenging assays were di(phenyl)-(2,4,6-trinitrophenyl)iminoazanium (DPPH^•^, Sigma-Aldrich), methanol (Synth), 2,2′-azino-bis(3-ethylbenzthiazoline-6-sulfonic acid) radicals (ABTS^•+^, Sigma-Aldrich), potassium persulfate (K_2_S_2_O_8_, Sigma-Aldrich), ethanol, sodium hypochlorite (NaOCl, Sigma-Aldrich), 3,3′,5,5′-tetramethyl[1,1′-biphenyl]-4,4′-diamine (TMB—Sigma-Aldrich), N,N-dimethylformamide (Synth), potassium iodide (Merck, Darmstadt, Germany), acetic acid (Merck), crocin (Sigma-Aldrich), 2,2′-azobis(2-amidinopropane) dihydrochloride (AAPH—Sigma-Aldrich) and dimethyl sulfoxide (Synth). 

### 3.2. Synthesis of Layered Zinc Hydroxide Nitrate Matrix

The layered host matrix (Zn-NO_3_-LSH) was prepared by the precipitation method [25]. A solution of NaOH (1.0 mol L^−1^) was slowly added to a zinc nitrate solution containing 1.75·10^−2^ mol L^−1^ of Zn^2+^ ions under nitrogen atmosphere and magnetic stirring, until the final pH adjusted to 6.5. The suspension was kept under magnetic stirring and nitrogen atmosphere during 3 h. The solid obtained was filtered, washed with deionized water, and dried in presence of silica at room temperature for 24 h.

### 3.3. Synthesis of LSH Intercalated with Ferulate Anions 

The LSH-ferulate materials were synthetized by the anion exchange method [23,26] and/or precipitation method at constant pH [25,26]. In the anion exchange method, the Zn-NO_3_-LSH matrix was dispersed in a solution of the sodium ferulate salt (C_10_H_9_O_4_^−^/Zn-NO_3_-LSH molar ratio equal to 23.5) and the suspension obtained had the final pH adjusted to 7 with addition of a 1.0 mol L^−1^ solution of NaOH. The suspension was subjected to magnetic stirring at room temperature for 5 days. The solid obtained was filtered, washed with deionized water and ethanol, and dried in presence of silica at room temperature for 24 h.

For LSH intercalated with ferulate anions obtained by the precipitation method at constant pH, a zinc nitrate solution was added slowly and under magnetic stirring to a solution of sodium ferulate salt (Zn^2+^/C_10_H_9_O_4_^−^ molar ratio equal to 3, 4, or 5) under nitrogen atmosphere. The solution pH was adjusted to 7 by the addition of a 1.0 mol L^−1^ solution of NaOH. The suspension obtained was kept under magnetic stirring for 3 days and subjected or not to ultrasound treatment (Unique^®^ ultrasonic cell disruptor, model DES500, ultrasound power of the 250 W) for 10, 20, or 30 min. The precipitated was filtered, washed with deionized water and ethanol, and dried in presence of silica at room temperature for 24 h.

### 3.4. Preparation of Cosmetic Formulations 

The cosmetic formulations were prepared by the colloidal dispersion of Nafel salt, Zn-NO_3_-LSH matrix or LSH-ferulate materials (Zn-fel(4)-LSH and Zn-fel(4)-LSH/U10 samples) in the oil phase of the skin care formulation described by Saito et al. [11]. The oil/water-type dispersions were subjected to magnetic stirring for 1 h to cream formation. The mass percentage of formulation ingredients and the cosmetic creams obtained are shown in Table 6.

### 3.5. Characterization Techniques

X-ray diffraction (XRD) patterns of powdered samples were recorded on a Rigaku diffractometer, model RINT 2000, using CuKα radiation (1.5418 Å, 40 kV, 70 mA, scan 2θ range 3–70°, and scan speed 0.02°/10 s) and Ni filter. Zeta potential measurements of layered materials dispersed in deionized water were collected in a Zetasizer Nano ZS analyzer (Malvern Instruments Ltd., Malvern, UK) using DTS 1070 cuvettes, dielectric constant equal to 78.5, and refractive and absorption index adjusted to 1.5 and 0.01, respectively. FTIR spectra were collected in the 4000–368 cm^−1^ region in a Bruker spectrophotometer, model FTIR Vertex70, using the ATR method. Color index was obtained in a Konica Minolta spectrophotometer, model CM-2500d, equipped with d/8° integrating sphere (CIELab color space). Diffuse reflectance spectra of powdered samples were recorded on a Perkin Elmer spectrophotometer, model LAMBDA 1050 UV–VIS–NIR, equipped with Spectralon 150 nm integrating sphere. Thermal analyses (TGA/DSC) of powered samples were recorded on a SQ600 thermoanalyzer (TA Instruments Inc. New Castle, USA) and a TGA 4000 thermoanalyzer (Perkin Elmer, Waltham, USA) using a heating rate of 10 °C min^−1^ and under synthetic air flow of 50 mL min^−1^.

### 3.6. Molecular Modeling Method

Molecular modeling of the ferulate anion was performed by semi-empirical calculations in the MOPAC2016 software, using the Sparkle/RM1 model with the keywords: BFGS, XYZ, SPARKLE, RM1, GNORM = 0.25, PRECISE and CHARGE = −1.

### 3.7. In Vitro Sun Protection Factor (SPF) Method 

The SPF values of cosmetic formulations were obtained from absorption spectra measurements using the *in vitro* sun protection factor assessment [51,52,64], which is defined by:

(1) $$SPF=\frac{\int_{290}^{400}E\left(\lambda \right)S\left(\lambda \right)d\lambda }{\int_{290}^{400}E\left(\lambda \right)S\left(\lambda \right)T\left(\lambda \right)d\lambda },$$

where *E*(*λ*) is the erythema action spectrum and *S*(*λ*) is the spectral irradiance of terrestrial sunlight under defined conditions by the International Organization for Standardization [65]. The *T*(*λ*) corresponds to the optical diffuse transmittance of cosmetic formulations as a function of wavelength (*λ*) and the wavelength integration limits refers to the combined UVB and UVA wavelength range. For the *in vitro* SPF assay, photoprotective films of cosmetic formulations were prepared by application of cosmetic creams on Transpore^®^ tape, which is a UV-transparent substrate [64]. The spread amount of cosmetic formulation on the Transpore^®^ substrate was 2.00 ± 0.1 mg cm^−2^ using doctor blade technique [66]. Absorption spectra of photoprotective films were recorded on a Perkin Elmer spectrophotometer, model LAMBDA 465 UV–VIS–NIR. The *in vitro* SPF measurements were realized in quadruplicate and SPF results were expressed as mean ± standard deviation ± confidence interval (confidence level of the 95%).

### 3.8. In Vitro Antioxidant Activity Assays

Spectrophotometric methods used to investigate the antioxidant activity of Nafel, Zn-NO_3_-LSH matrix, and LSH-ferulate materials (Zn-fel(4)-LSH and Zn-fel(4)-LSH/U10 samples) were capture methods of di(phenyl)-(2,4,6-trinitrophenyl)iminoazanium (DPPH^•^) and 2,2′-azino-bis(3-ethylbenzthiazoline-6-sulfonic acid) (ABTS^•+^) radicals, hypochlorous acid (HOCl/OCl^−^) scavenging assay and crocin bleaching assay. It is important to emphasize that these reactive species assays were realized in duplicate and capture (inhibition) percentage of reactive species was calculated from test results using the equation:

(2) $$I=\left(\frac{A_{0}-A_{c}}{A_{0}}\right),$$

where *I* is the capture (inhibition) percentage of reactive species, *A*_0_ corresponds to absorbance of the chromophore compound in the absence antioxidant, and *A_c_* is the absorbance of the chromophore compound in presence of the antioxidant sample. The absorbance measurements were recorded on a microplate spectrophotometer (Biotek-Power Wave XS2, Winooski, VT, USA) and an OceanOptics USB 4000 for the tests with crocin, with magnetic stirring and Peltier heating.

#### 3.8.1. Method of DPPH^•^ Radical Capture

According to the methodology described by Soares et al. [67], DPPH assay is based on the absorbance monitoring of DPPH^•^ radicals at 517 nm, after incubation in the dark at 25 °C for 20 min, in the absence and presence of ethanolic solutions of samples. Inhibition percentage was calculated by average absorbance of DPPH^•^ species as function of samples concentrations.

#### 3.8.2. ABTS Radical Cation Decolorization Assay

The antioxidant activity of samples was assessed by ABTS^•+^ radical cation decolorization assay described by Re et al. [68] with modifications. The ABTS^•+^ radicals were produced by oxidation of the 2,2′-azino-bis(3-ethylbenzthiazoline-6-sulfonic acid) (7.0 × 10^−3^ mol L^−1^) with K_2_S_2_O_8_ oxidizing agent (0.14 mol L^−1^) in the absence of light at room temperature for 16 h. The ABTS^•+^ stock solution was diluted in sodium phosphate buffer (0.01 mol L^−1^ and pH = 7.0) to an absorbance of 0.750 ± 0.020, at 734 nm. Different amounts of the sample were added to ABTS^•+^ solution and the reaction mixtures were incubated for 15 min in the dark at room temperature. Absorbance values of these mixtures were monitored at 734 nm and inhibition percentage was calculated by average absorbance of ABTS^•+^ species as function of samples concentrations. 

#### 3.8.3. Hypochlorous Acid (HOCl/OCl^−^) Scavenging Assay

In this assay, antioxidant activity is directly associated to interactions between sample and HOCl/OCl^−^ species, which prevents the formation of the blue chromophore compound produced by oxidation of the 3,3′,5,5′-tetramethyl[1,1′-biphenyl]-4,4′-diamine (TMB). This blue chromophore compound has maximum absorbance at 655 nm [57]. A standard solution of OCl^−^ was prepared by dissolution of the NaOCl in a NaOH solution (0.01 mol L^−1^) and the concentration of this standard solution was obtained by its molar absorptivity coefficient (ε = 350 L mol^−1^ cm^−1^ at 292 nm) [69]. Various concentrations of samples in sodium phosphate buffer (0.05 mol L^−1^ and pH = 7.4) were incubated with HOCl/OCl^−^ (3.0 × 10^−5^ mol L^−1^) for 10 min. TMB (2.8·× 10^-3^ mol L^−1^ dissolved in 50% N,N-dimethylformamide with 0.01 mol L^−1^ potassium iodide in 0.8 mol L^−1^ acetic acid) was then added and incubated for 5 min at room temperature in the dark and the absorbance were monitored at 655 nm, as described by Dypbukt [70], with modifications. The assay without the sample was used as the control (100% reaction) and the absorbance of the reaction medium without HOCl was used as a reading blank. The inhibition percentage was calculated by average absorbance of the TMB chromophore in presence of HOCl/OCl^−^ species as function of sample concentrations.

#### 3.8.4. Crocin Bleaching Assay

According to the methodology described by Tubaro et al. [71], crocin bleaching assay is based on the absorbance monitoring of the crocin at 443 nm for 10 min in a competitive kinetics procedure. The thermolysis reaction of the 2,2′-azobis(2-amidinopropane) dihydrochloride (AAPH) at 40 °C produces peroxyl radicals (ROO^•^) at a constant rate, an aerated medium [63]. Thus, a kinetic competition for capturing of ROO^•^ species occurs between crocin and antioxidants; consequently, the inhibition depends on the ability of samples in capture of these radicals [57,63]. In this test, a crocin stock solution was prepared by dissolution of the crocin in dimethyl sulfoxide (DMSO) and its concentration was obtained by its molar absorptivity coefficient (ε = 13.726 L mol^−1^ cm^−1^ at 443 nm) [63]. The crocin solution (2.5·× 10^−5^ mol L^−1^) and sample solutions with different concentrations were added in a sodium phosphate buffer (0.12 mol L^−1^ and pH = 7.0) resulting in a mixture solution. The thermolysis reaction was started by addition of the AAPH solution (12.5·× 10^−3^ mol L^−1^) in the mixed solution under constant stirring at 40 °C. The rate of crocin bleaching (linear behavior after 100 s of the reaction) was monitored at 443 nm for 10 min. In order to eliminate possible interference of the sample, a test without crocin was performed for each solute and used as blank reaction. The crocin bleaching rate (*V*_0_) decreases in the presence of an antioxidant compound due to kinetic competition between crocin and antioxidant for capture to ROO^•^ radicals. Therefore, the new bleaching rate (*V*) is given by:

(3) $$V=V_{0}\times \frac{k_{c}\left[C\right]}{k_{c}\left[C\right]+k_{A}\left[A\right]},$$

where *V*_0_ = *k*_1_[ROO^•^][*C*], *k_c_* = *k*_1_[ROO^•^], *k_a_* = *k*_2_[ROO^•^], [ROO^•^] is a concentration of the peroxyl radical, *V*_0_ corresponds to reaction between crocin and ROO^•^ species, *k*_1_ and *k*_2_ are rate constants for the ROO^•^—crocin reaction and ROO^•^—sample reaction, [*C*] is the crocin concentration, and [*A*] is the sample concentration. The decrease of the crocin bleaching rate in the presence of an antioxidant can be described by:

(4) $$\frac{V_{0}}{V}=\frac{k_{c}\left[C\right]+k_{A}\left[A\right]}{k_{c}\left[C\right]}=1+\frac{k_{A}}{k_{c}}\times \frac{\left[A\right]}{\left[C\right]}.$$

The *k_A_/k_c_* constant value corresponds to the angular coefficient obtained by linear regression for *V*_0_/*V versus* [*A*]/[*C*] graph and indicates the relative ability of an antioxidant to interact with ROO^•^ radicals. By dividing the *k_A_*/*k_c_* constant of the sample by *k_A_*/*k_c_* constant of a standard antioxidant, such as Trolox, it is possible to obtain the ratio of these constants and relative antioxidant capacity of the analyzed compound [57,63]. 

## 4. Conclusions

LSH-ferulate materials were successfully synthesized by the anion exchange method and precipitation method at constant pH varying Zn^2+^/fel^−^ molar ratio and ultrasound treatment time. Structural characterization shows the formation of layered materials composed by a mixture of LSH intercalated with ferulate anions. The intercalation process of ferulate anions in the LSH-ferulate materials is proven by increase of the basal spacing noted in XRD patterns, presence and changes of characteristic vibrational bands of ferulate anion, and LSH host observed in the FTIR and modifications of UV-VIS absorption bands when compared to Nafel salt and the Zn-NO_3_-LSH matrix. According to the interlayer spacing values of layered materials, ferulate anion dimensions, and host–guest interactions observed in the XRD, FTIR, and UV-VIS results, mono and/or bilayer arrangements of organic anions in the interlayer region are proposed. In addition, LSH-ferulate samples exhibit yellowish-white color that do not compromise their use in the cosmetic products.

The precipitation method at constant pH advantages the interactions of the ferulate anion in the LSH host. Nevertheless, the anion exchange method gives rise to a layered material with an excess amount of adsorbed ferulate anions as seen in the zeta potential and FTIR results. The cavitation effect produced by ultrasound treatment causes structural changes in the LSH-ferulate samples, which results in an emergence of LSH phases, modifications in vibrational and UV-VIS absorption bands, and different chemical composition observed in TGA/DSC results. Moreover, the intercalation of ferulate anions in the LSH host provides the increase of the thermal stability of these organic species.

UV-VIS absorption spectra and *in vitro* SPF values indicate that LSH-ferulate materials present UV shielding ability, mainly UVB protection. Moreover, reactive species assay results show that layered materials have capacity to capture DPPH^•^, ABTS^•+^, ROO^•^, and HOCl/OCl^−^ reactive species, consequently, they have potential to reduce damages to the human organism caused by reactive species effects. Optical properties combined to antioxidant activity of LSH-ferulate samples provide simultaneous beneficial functions to the human organism. Therefore, LSH-ferulate materials have singular properties that allow their use as multifunctional filters. 

## Figures and Tables

**Table 1:** Interplanar distance d_200_, basal distance, and interlayer spacing of LSH materials.

Sample	d_200_/Å	Basal Distance/Å	Interlayer Spacing/Å
Zn-NO_3_-LSH	9.74	9.74	-
Zn-fel-LSH/A	26.30	26.17	16.17
	13.08 *	13.05 *	3.05 *
Zn-fel(3)-LSH	27.10	26.54	16.54
	14.35 *	14.48 *	4.48 *
	13.27 ^#^	13.19 ^#^	3.19 ^#^
Zn-fel(4)-LSH	26.94	26.45	16.45
	13.23 *	13.17 *	3.17 *
Zn-fel(4)-LSH/U10	26.94	26.48	16.48
	15.40 *	15.33 *	5.33 *
	13.31 ^#^	13.23 ^#^	3.23 ^#^
Zn-fel(4)-LSH/U20	25.83	25.99	15.99
	14.98 *	15.14 *	5.14 *
	13.00 ^#^	13.03 ^#^	3.03 ^#^
Zn-fel(4)-LSH/U30	26.61	26.44	16.44
	15.29 *	15.28 *	5.28 *
	13.27 ^#^	13.17	3.17
Zn-fel(5)-LSH	26.77	26.37	16.37
	15.24 *	15.27 *	5.27 *
	13.15 ^#^	13.12 ^#^	3.12 ^#^

*,^#^ Different LSH phases.

**Table 2:** Frequencies of the carboxylate asymmetric and symmetric stretching modes and frequency difference values (Δν = ν_as_ − ν_s_) between carboxylate stretching modes of the sodium ferulate salt (Nafel—NaC_10_H_9_O_4_) and LSH-ferulate materials.

Sample	ν_as_/cm^−1^	ν_s_/cm^−1^	Δν (ν_as_ − ν_s_)/cm^−1^
Nafel	1539	1379	160
Zn-fel-LSH/A	1560	1400	160
	1508		108 *
Zn-fel(3)-LSH	1562	1394	168
	1510		116 *
Zn-fel(4)-LSH	1560	1394	166
	1510		116 *
Zn-fel(4)-LSH/U10	1562	1390	172
	1508		118 *
Zn-fel(4)-LSH/U20	1562	1398	164
	1510		112 *
Zn-fel(4)-LSH/U30	1562	1391	171
	1508		117 *
Zn-fel(5)-LSH	1562	1394	168
	1510		116 *

* Different coordination mode assigned to the same LSH-ferulate material.

**Table 3:** Mass percentage (wt%) attributed to water loss, ferulate decomposition, and residue formed by thermal decomposition of the Zn-fel(4)-LSH and Zn-fel(4)-LSH/U10 samples.

Sample	H_2_O/wt%	Ferulate Anion/wt%	Residue/wt%
Zn-fel(4)-LSH	18	35	47
Zn-fel(4)-LSH/U10	38	19	43

**Table 4:** *In vitro* SPF values of commercial sunscreen product, base, Zn-NO_3_-LSH, Zn-fel(4)-LSH/U10, Nafel/1, Zn-fel(4)-LSH, and Nafel/2 creams.

Formulation	SPF	Standard Deviation	Confidence Interval—95%
Commercial sunscreen *	8.6	2.4	3.8
Base	2.3	0.4	0.7
Zn-NO_3_-LSH	7.8	0.7	1.1
Zn-fel(4)-LSH/U10	8.0	1.5	2.3
Nafel/1	6.8	0.7	1.2
Zn-fel(4)-LSH	12.1	0.9	1.4
Nafel/2	7.0	1.1	1.8

* Commercial sunscreen product that has SPF labeled equal to 10.

**Table 5:** DPPH^•^, ABTS^•+^, HOCl/OCl^−^, and ROO^•^ scavenging activities of Nafel (NaC_10_H_9_O_4_), Zn-fel(4)-LSH and Zn-fel(4)-LSH/U10 samples expressed in the EC_50_ values and linear regression slopes for the V_0_/V versus [Antioxidant]/[Crocin] graphs.

Sample	DPPH^•^	ABTS^•+^	HOCl/OCl^−^	ROO^•^	Slope
	EC_50_/mg mL^−1^	EC_50_/mg mL^−1^	EC_50_/mg mL^−1^	EC_50_/mg mL^−1^	
Nafel	1.34	0.00101	0.000827	0.00615	4.93
Zn-fel(4)-LSH	0.0882	0.00615	0.00297	0.170	0.26
Zn-fel(4)-LSH/U10	0.0957	0.00504	0.00319	0.209	0.16

**Table 6:** Mass percentage of formulation ingredients of cosmetic creams containing Zn-NO_3_-LSH, Zn-fel(4)-LSH, Zn-fel(4)-LSH/U10, or Nafel (NaC_10_H_9_O_4_) compound.

Ingredients	Phase	Cosmetic Formulations/%					
Cetostearyl alcohol	Oil	2.00	2.00	2.00	2.00	2.00	2.00
2,3-dihydroxypropyl octadecenoate	Oil	2.00	2.00	2.00	2.00	2.00	2.00
Cosmowax^®^ J.	Oil	8.00	8.00	8.00	8.00	8.00	8.00
Dipropan-2-yl hexanedioate	Oil	1.50	1.50	1.50	1.50	1.50	1.50
(1-decanoyloxy-3-octanoyloxypropan-2-yl)dodecanoate	Oil	1.50	1.50	1.50	1.50	1.50	1.50
Zn-NO_3_-LSH	Oil	-	5.00	-	-	-	-
Zn-fel(4)-LSH	Oil	-	-	5.00	-	-	-
Zn-fel(4)-LSH/U10	Oil	-	-	-	5.00	-	-
Nafel	Oil	-	-	-	-	1.8	0.1
Propane-1,2-diol	Aqueous	4.00	4.00	4.00	4.00	4.00	4.00
Methyl 4-hydroxybenzoate	Aqueous	0.18	0.18	0.18	0.18	0.18	0.18
Propyl 4-hydroxybenzoate	Aqueous	0.02	0.02	0.02	0.02	0.02	0.02
2,2′,2″,2‴-(1,2-Ethanediyldinitrilo)tetraacetic acid	Aqueous	0.05	0.05	0.05	0.05	0.05	0.05
Distilled Water	Aqueous	80.75	75.75	75.75	75.75	78.95	80.65

## Sample Availability

Samples of the layered compounds, Zn-NO_3_-LSH, Zn-fel-LSH/A, Zn-fel(3)-LSH, Zn-fel(4)-LSH, Zn-fel(5)-LSH, Zn-fel(4)-LSH/U10, Zn-fel(4)-LSH/U20 and Zn-fel(4)-LSH/U30, are available from the authors.
